# Development of a homogeneous liquid–liquid microextraction method using lighter than water solvents for the extraction of some pesticides from distillate samples followed by GC-FID determination

**DOI:** 10.1039/d5ra07135f

**Published:** 2025-11-18

**Authors:** Mahsa Ghoreishizadeh, Mir Ali Farajzadeh, Sanaz Barazandeh, Mohammad Reza Afshar Mogaddam

**Affiliations:** a Department of Analytical Chemistry, Faculty of Chemistry, University of Tabriz Tabriz Iran mafarajzadeh@yahoo.com mafarajzadeh@tabrizu.ac.ir +98 41 33340191 +98 41 33393084; b Engineering Faculty, Near East University 99138 Nicosia, North Cyprus Mersin 10 Turkey; c Food and Drug Safety Research Center, Pharmaceutical Sciences Institute, Tabriz University of Medical Sciences Tabriz Iran; d New Material and Green Chemistry Research Center, Khazar University 41 Mehseti Street Baku AZ1096 Azerbaijan; e Pharmaceutical Analysis Research Center, Pharmaceutical Sciences Institute, Tabriz University of Medical Sciences Tabriz Iran

## Abstract

In this work, an efficient sample preparation method for the extraction and preconcentration of some pesticides from different distillate samples based on a homogeneous liquid–liquid microextraction method was developed. For this aim, in a glass test tube specially designed with a capillary tip, a standard solution of the analytes along with a few µL of *n*-hexanol was added and vortexed to obtain a homogeneous solution. Then, µL-volume of di-*n*-butyl ether was added. After vortexing, a turbid solution was obtained. After centrifuging, the organic phase, including the extracted analytes, was collected at the beginning of the capillary part of the tube. Then, an aliquot of the organic phase was injected into a gas chromatograph equipped with a flame ionization detector. Different parameters, including solvent type, solvent volume, centrifuging time, vortexing time, and pH, were optimized. In this work, high enrichment factors (160–662) and well-linear calibration curves (*r*^2^ = 0.986–0.999) were obtained. The limits of detection and quantification were obtained in the ranges of 0.11–0.55 and 0.35–1.83 µg L^−1^, respectively. The precision of the procedure was examined *via* relative standard deviation for both intra-day (*n* = 7) and inter-day (*n* = 3) repeatabilities. Intra- and inter-day repeatabilities at a concentration of 25 µg L^−1^ (for each pesticide) were obtained in the ranges of 3.6–13.2% and 5.8–13.3%, respectively. Intra- and inter-day repeatabilities at the concentration of 100 µg L^−1^ were obtained as 3.9–7.7% and 5.1–9.9%, respectively. The useful points of this method were a short extraction time, simple implementation, low cost, and environmental compatibility.

## Abstract 2

Development of an efficient sample preparation method for the extraction and preconcentration of some pesticides from different distillate samples based on a homogeneous liquid–liquid microextraction method.

## Graphical abstract

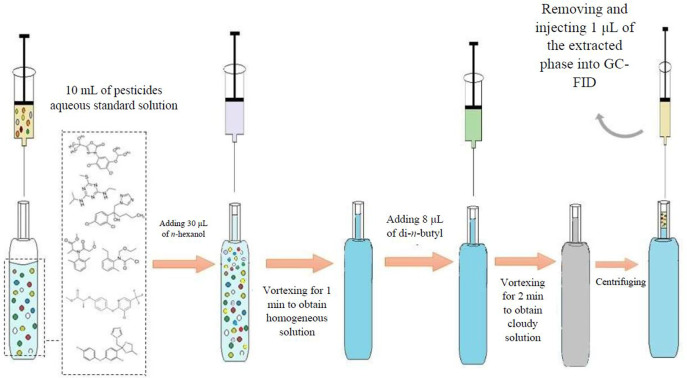

## 1. Introduction

Pesticides are widely applied nowadays. These compounds are used to eliminate various pests, and without their use, agricultural and food productions significantly reduce. Therefore, pesticides are used to achieve high output and ensure food safety by controlling pests and weeds.^1^ They are persistent and cannot be removed. They can enter the food chain and cause serious harm to humans by causing various diseases such as asthma, cancers, Parkinson's disease, and endocrine-related diseases.^2,3^ As a result, regular measurement of the amount of these compounds in water and food sources is very important.^4^ For each pesticide, the World Health Organization has announced a permissible amount. There are various methods, such as electrophoresis,^5^ spectrophotometry,^6^ and chromatography,^7^ for the detection and quantification of pesticides. Problems such as low concentration and matrix complexity can hinder the direct analysis of pesticides, thereby reducing the sensitivity of the analysis and enhancing the limits of detection (LOD) and quantification (LOQ).^8^ Therefore, appropriate sample preparation methods should be used to address these problems.^8,9^ Homogeneous liquid–liquid extraction (HLLE) is a preconcentration method for contaminants from complex matrices. HLLE was introduced in 1973 for the extraction of polar organic compounds and biological materials.^10^ In 2009, the homogeneous liquid–liquid microextraction (HLLME) method was proposed for the first time.^11^ HLLME and dispersive liquid–liquid microextraction (DLLME) are very similar. Among them, DLLME is widely used by researchers.^12,13^ DLLME is a method with a ternary solvent system containing an aqueous phase, an extraction solvent, and a disperser solvent.^14^ During this extraction method, extraction solvents with a high affinity towards analytes and low solubility in water are chosen. It should also form fine droplets in the presence of the dispersion solvent.^15^ DLLME has many advantages, such as great EFs, short extraction time, and low operating cost. However, it has some limitations, such as introducing relatively high contamination from organic solvents, especially from the dispersive solvent.^16^ HLLME consists of a binary solvent system (aqueous phase and extraction solvent); therefore, less contamination by organic solvents is caused compared to DLLME.^17^ In HLLME, hydrophilic organic solvents that are partially soluble (*n*-hexanol, butanol, and isobutanol) or completely soluble (acetone, acetonitrile, ethanol, *etc.*) in water are used as the extraction solvents.^18^ HLLME is a relatively simple and fast method with high EFs;^19^ it consumes less sample and organic solvents and is a simple process that does not require a dispersive solvent.^20^ Recent advances in the treatment of pesticide-containing wastewater (*e.g.*, full-spectrum photocatalytic degradation and catalyst-based oxidation pathways) have demonstrated the increasing ability to degrade organophosphates and other pesticides. However, these developments also underscore the need for sensitive and low-loss analytical tools to monitor the main residual compounds and degradation products during and after treatment.^21^ As discussed herein, such progress has further motivated the development of microextraction methods with minimal organic solvent usage.

In this method, a homogeneous solution is formed in which there is no barrier between the aqueous phase and the organic phase (extraction solvent).^22^ The absence of a barrier that prevents surface contact between the two phases during the extraction process allows this method to be very fast. The extraction solvent must have strong interactions with the target analytes to extract them from the complex matrix.^23,24^ This method is commonly used for the extraction and analysis of various analytes.^25^ HLLME is performed in various ways, and the addition of external agents such as salt^26^ or sugar,^27^ changing the temperature (cooling or heating)^28^ and pH,^29^ and use of another solvents^30^ can break the homogeneity. Several homogeneous liquid–liquid microextraction methods have been previously developed for the extraction of pesticides from environmental and food samples. For instance, classical ternary solvent HLLME methods often require the use of salting-out agents or pH adjustment to induce phase separation, which complicates the procedure and affects reproducibility.^31^ Ultrasound-assisted salting-out HLLME has been applied with triazole pesticides, but it needs ultrasonic equipment and careful control of the salt concentration.^32^ Other studies have employed chlorinated solvents such as chloroform for organophosphorus pesticide extraction, thus raising safety and environmental concerns.^33^ Flotation-assisted HLLME eliminates centrifugation but shows poor phase stability and limited enrichment for complex matrices.^34^ Compared with these techniques, the present method, using *n*-hexanol and di-*n*-butyl ether, offers simpler operation, requires no external phase-separation agent or ultrasonic assistance, minimizes solvent consumption, and facilitates easy phase collection, resulting in a more efficient and eco-friendly sample preparation procedure. Various microextraction strategies have been reported to improve extraction efficiency and reduce solvent consumption, such as salting-out assisted liquid–liquid extraction.^35^ This approach demonstrates the potential of manipulating ionic strength to promote phase separation, inspiring the development of more environmentally friendly and efficient HLLME techniques such as that proposed in this study.

Herein, an attempt has been made to break the homogeneity by using another solvent, and it has been applied in the extraction and preconcentration of some pesticides from various distillates. In a specially designed test tube with a capillary tip, aqueous solution of the analytes or distillate was added. Di-*n*-butyl ether was used as the homogeneity-breaking agent. *n*-Hexanol was the solvent used to obtain a homogeneous solution. Due to the low density of the solvents used compared to water, the extractive phase was collected on top of the aqueous phase, then an aliquot of the extract was injected into a gas chromatography-flame ionization detector (GC-FID) system for quantitative analysis. Various parameters of the extraction method were carefully optimized.

## 2. Experimental

### 2.1. Chemicals and solutions

Pesticides with purity of over 98% were obtained from the Dr Ernsthofer Company (Augsburg, Germany), including acetochlor, metalaxyl, ametryn, haloxyfop-*R*-methyl, hexaconazole, oxadiazon, triticonazol, and difenoconazole. To prepare a stock solution (1000 mg L^−1^ of each pesticide), appropriate amounts of each pesticide were dissolved in methanol. To prepare the aqueous standard solution used in the extraction procedure, this solution was diluted with deionized water. Another standard solution of pesticides was prepared in di-*n*-butyl ether (1000 mg L^−1^ of each), having 200 mg L^−1^ of cetyl alcohol as the internal standard. It was injected three times each day, and the peak areas for pesticides and cetyl alcohol were used in the calculation of EFs. Di-*n*-butyl ether and *n*-hexanol, which were used as organic solvents for HLLME, were procured from Merck (Darmstadt, Germany). *n*-Hexane, toluene, and xylene were also purchased from Merck. Potassium chloride, sodium sulphate, and sodium chloride, also from Merck, were used in the optimization step. Robinson buffer was used to adjust the pH, in which the boric acid, phosphoric acid, and acetic acid were procured from Merck, and sodium hydroxide was procured from Sigma (St. Louis, MO, USA). Cetyl alcohol from Fluka (Seelze, Germany) was used as an internal standard.

### 2.2. Samples

Five types of distillates, including cardamom, cinnamon, mint, pussy willow, and Moldavian dragonhead, were obtained from a native store (Tabriz, Iran). The real samples mentioned were diluted at a 1 : 1 ratio with deionized water before use.

### 2.3. Apparatus

The chromatographic apparatus was a Shimadzu gas chromatograph (model 2014, Kyoto, Japan) equipped with an FID and a split/splitless injection system. The following temperature program was used for the differentiation of analytes. At the outset, the column oven was set at 60 °C for 1 min and then enhanced to 300 °C with a rate of 10 °C min^−1^ and kept for 1 min. An RTX-5 capillary column (column length 30 m × 0.25 mm id × 0.25 µm film thickness) was applied for the differentiation of analytes. Helium (99.999%) (Gulf Cryo, Dubai, UAE) was used as the carrier gas at a stable linear velocity of 30 cm s^−1^ and as the make-up gas at a flow rate of 40 mL min^−1^. Air was used as the oxidant in the FID at a flow rate of 300 mL min^−1^. A Shimadzu hydrogen generator (OPGU-1500S) was applied to produce hydrogen at a flow rate of 30 mL min^−1^. A 1 µL microsyringe (Hamilton, Bonaduz, Switzerland) was used to inject the standard solutions and extracts into the separation system. A model 654 pH meter (Herisau, Switzerland) was used to adjust the pH, and a Hettich D-7200 centrifuge D-7200 centrifuge (Kitchener, Germany) was used to separate the phases. An L46 vortex (Labinco, Breda, the Netherlands) was applied to vortex the solutions.

### 2.4. Extraction procedure

**Fig. 1:**
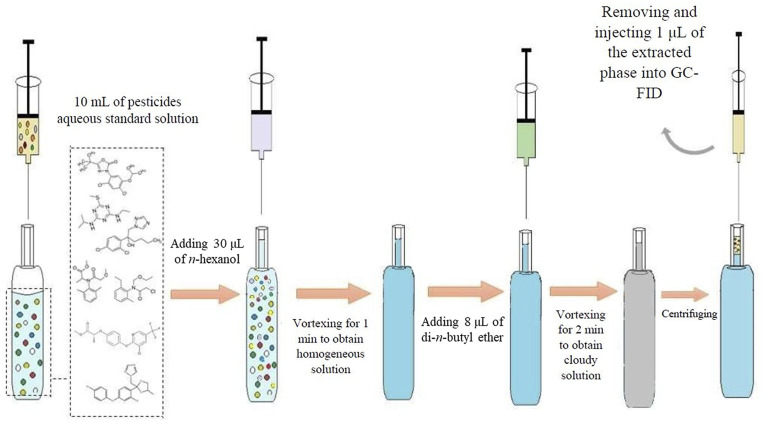
Schematic of the extraction process.

To a glass test tube with a capillary top, 10 mL of sample or standard solution of pesticides with a concentration of 200 µg L^−1^ (of each) was added. After that, 30 µL of *n*-hexanol was added and vortexed for 1 min to obtain a homogeneous solution. Then, 8 µL of breaking solvent (di-*n*-butyl ether) containing an internal standard (cetyl alcohol at a concentration of 200 mg L^−1^) was added. After vortexing for 2 min, a cloudy solution was obtained. After centrifuging for 5 min at a rate of 7000 rpm, the analytes were extracted into the organic phase and collected above the water (due to the low density of the organic solvents) in the capillary section of the tube. One microliter of the extractive phase was injected into the differentiation system for quantitative analysis. The extraction method is schematically displayed in Fig. 1.

### 2.5. Calculation of EF

In this study, the collected organic phase was very low, at the level of a few µL. To obtain good repeatability in the analytical data, cetyl alcohol was used as an internal standard to assist with the volume of the extract. In the implementation of the method, the internal standard was used at a concentration of 200 mg L^−1^ in the breaking solvent (di-*n*-butyl ether), and EF was calculated from eqn (1). In this equation, *A*_anal_ represents the peak area of the pesticide in the extract. *A*_Is_ and *C*_Is_ represent the peak area and concentration (mg L^−1^) of the internal standard, respectively. *C*_0_ indicates the concentration of analyte (mg L^−1^) in the initial aqueous phase.1
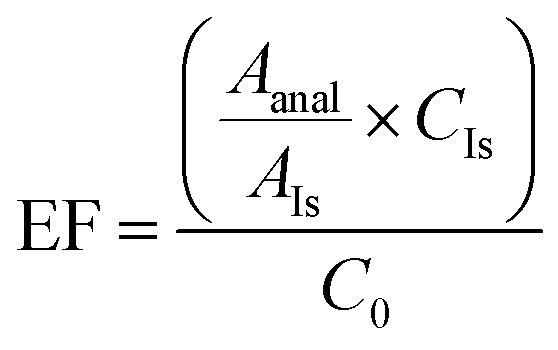


## 3. Results and discussion

### 3.1. Homogeneity-breaking solvent type

**Fig. 2:**
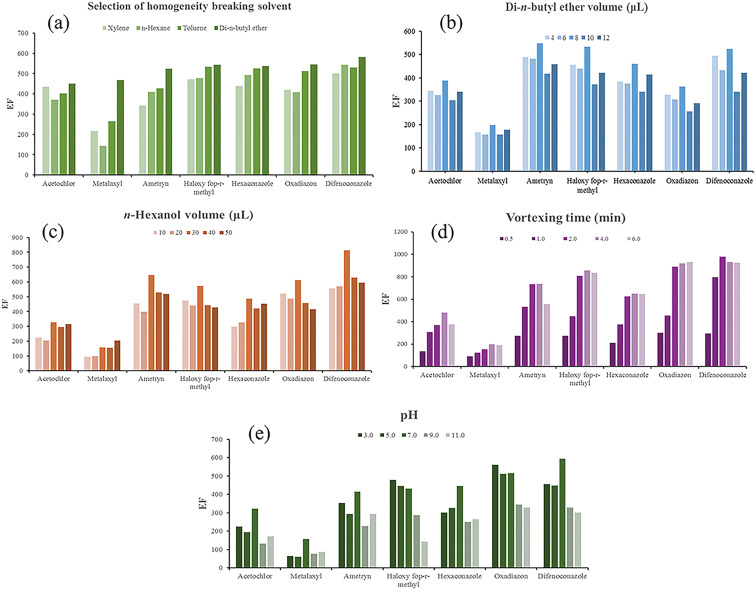
Optimization of the experimental parameters affecting the extraction efficiency of the proposed HLLME method: (a) type of the homogeneity-breaking solvent. Extraction conditions: HLLME procedure: aqueous solution volume, 10 mL of deionized water spiked with 200 µg L^−1^ of each analyte; solvent creating a homogeneous phase (volume), *n*-hexanol (50 µL); vortexing time, 5 min; centrifugation rate, 7000 rpm, and centrifugation time, 5 min. (b) Volume of di-*n*-butyl ether. Extraction conditions: Similar to the conditions mentioned in (a), except that di-*n*-butyl ether was used as the homogeneity-breaking solvent. (c) *n*-Hexanol volume. Extraction conditions: similar to the conditions mentioned in (b), except that 8 µL of di-*n*-butyl ether was used. (d) Vortexing time. Extraction conditions: similar to the conditions mentioned in (c), except that 30 µL of *n*-hexanol was used. (e) pH. Extraction conditions: similar to the conditions mentioned in (d), except that vortexing was performed for 2 min.

Initially, a microliter amount of *n*-hexanol was added to an aqueous solution of the analyte. The volume of *n*-hexanol was very low, and a homogeneous solution was obtained. By adding another organic solvent at the µL-level (homogeneity-breaking solvent), a turbid solution was achieved by dispersion of the organic phase into the aqueous phase with the aid of *n*-hexanol. The choice of the homogeneity-breaking solvent is important. The basis of this work was the collection of the organic phase above the aqueous phase. For this purpose, solvents with a density lower than water were selected. By adding the homogeneity-breaking solvent, a cloudy state should be created in the mixture of water and *n*-hexanol. To rationalize the solvent selection, Hansen solubility parameters (*δ*D, *δ*P, *δ*H) and density/surface tension data for the tested low-density solvents were obtained from the HSPiP/Hansen database (*δ* values in MPa^0.5^) (toluene: *δ*D 18.0; *δ*P 1.4; and *δ*H 2.0, xylene: *δ*D 17.8; *δ*P 1.0; and *δ*H 3.1, *n*-hexane: *δ*D 14.9; *δ*P 0.0; and *δ*H 0.0, and di-*n*-butyl ether: *δ*D 15.2; *δ*P 3.4; and *δ*H 4.2). Among these, toluene, xylene, di-*n*-butyl ether, and *n*-hexane were selected as light solvents. These data indicate that di-*n*-butyl ether has the most favorable combination of dispersive/polar/hydrogen bonding components and surface properties for the extraction of the studied pesticides under HLLME conditions. Thus, the Hansen solubility parameters approach provides a quantitative basis for the observed extraction performance and complements the experimental optimization results. According to the results in Fig. 2a, di-*n*-butyl ether was chosen as the optimum homogeneity-breaking solvent.

### 3.2. Optimization of di-*n*-butyl ether volume

In this step, the volume of *n*-hexanol was kept constant (50 µL), and the volume of di-*n*-butyl ether was optimized. Considering the available EFs for the volumes of 4, 6, 8, 10, and 12 µL, 8 µL of di-*n*-butyl ether was selected as the optimal volume. The results are shown in Fig. 2b. By enhancing the volume of di-*n*-butyl ether from 4 to 8 µL, the obtained EFs increased. However, at higher volumes (10 and 12 µL), the dilution effect reduced the EFs. The volume of the breaking solvent controls a trade-off between the available extraction capacity and dilution of the extract. Small increases in volume increase the total mass extracted, but beyond an optimum volume, the analyte concentration in the collected organic phase is reduced due to the dilution effect, leading to reduced EF. This explains the observed maximum EFs at 8 µL.

### 3.3. Optimization of *n*-hexanol solvent volume

*n*-Hexanol was used as the solvent to obtain a homogeneous solution of the aqueous phase and the organic solvent. This solvent has relatively good solubility in water (5900 mg L^−1^) and, therefore, the volume of this solvent was optimized. Volumes of 10, 20, 30, 40, and 50 µL of *n*-hexanol were investigated. The obtained EFs increased from 10 to 30 µL and were then reduced at higher volumes (30 µL) owing to the dilution effect. *n*-Hexanol acted as the homogenising solvent (partial water miscibility). At low volumes, droplet formation is insufficient for mass transfer; at high volumes, the aqueous phase becomes richer in organic co-solvent, reducing the partition coefficients and thus EFs. The observed optimum at 30 µL is consistent with these competing effects. Therefore, 30 µL of *n*-hexanol was selected as the optimal volume due to the higher EFs obtained (Fig. 2c).

### 3.4. Vortexing time optimization

Vortexing was used to accelerate the extraction in this study. For this purpose, vortexing times of 0.5, 1.0, 2.0, 4.0, and 6.0 min were investigated. At the 4.0 min vortexing time, EFs increased to the maximum values and then remained nearly constant at 6.0 min vortexing time. However, the repeatability of the residual volume at 4.0 min was low and unreliable. Moreover, at 6.0 min vortexing time, the volume of the collected organic phase decreased and its handling was difficult. Therefore, according to Fig. 2d, a vortex time of 2.0 was selected as the optimum time of vortexing for this step. Vortexing provides convective mass transfer and droplet dispersion. An intermediate time (2 min) produced sufficient dispersion for rapid extraction while avoiding droplet coalescence and reducing the phase recovery observed at longer vortexing times.

### 3.5. pH effect

The extraction process and stability of analytes in the aqueous phase may be affected by pH. To investigate this parameter, pH values of 3.0, 5.0, 7.0, 9.0, and 11.0 were adjusted by adding Robinson buffer (0.04 M). In this study, EF values were reduced in acidic and basic pH for most analytes, as illustrated in Fig. 2e. The pH influenced extraction mainly through changes in the analyte ionization state. Neutral species partition more readily into the organic phase. For the pesticides studied, neutral forms dominate at near-neutral pH, which explains the observed maximum EFs around pH 7; therefore, pH 7.0 was chosen as the optimum.

### 3.6. Centrifuging time and rate

Centrifugation times of 3, 5, and 7 min were evaluated. According to the results, 5 min was selected as the optimum centrifuging time. The rates of 4000, 5000, 6000 and 7000 rpm at the constant centrifuging time (5 min) were tested. The rate of 7000 rpm was selected as optimal, according to the data obtained (data not shown here). Centrifugation promotes coalescence and separation of the dispersed organic phase. The selected conditions (7000 rpm for 5 min) were empirically optimal for the rapid and reproducible recovery of the µL-scale organic layer without entrainment of aqueous microdroplets.

Among all the examined variables, the type of extraction solvent, pH of the aqueous phase, and organic solvent volume were identified as the key factors governing extraction efficiency. The solvent's polarity and Hansen solubility parameters determine the distribution coefficient of analytes, while pH controls the ionization state of pesticides and thus their hydrophobicity. The organic phase volume affects both the partitioning ratio and dilution of the analytes. Agitation and centrifugation mainly influence mass-transfer kinetics and phase separation. These combined effects explain the observed extraction trends and justify the optimized conditions selected for this study.

### 3.7. Method validation

To investigate the method efficiency, some characteristics such as the coefficient of determination (*r*^2^), linear range (LR) of the calibration curves, EF, relative standard deviation (RSD), LOD, and LOQ were calculated and reported in Table 1. For the computation of LODs and LOQs, the signal-to-noise ratios of 3 and 10 were considered, respectively. LODs were obtained in the range of 0.11–0.55 µg L^−1^, and LOQs were obtained in the range of 0.35–1.83 µg L^−1^. The *r*^2^ values were achieved between 0.986 and 0.999. EFs were in the range of 160–662. The RSD amounts for examining intra- (*n* = 7) and inter-day (*n* = 3) precisions at the concentration of 25 µg L^−1^ were in the ranges of 3.6–13.2% and 5.8–13.3%; at the concentration of 100 µg L^−1^, they were 3.9–7.7% and 5.1–9.9%, respectively.

**Table 1:** Quantitative features of the developed analytical method for the analysis of pesticides

Analyte	LOD^a^	LOQ^b^	LR^c^	*r* ^2^ d	eRSD% at the concentrations of				EF ± SD^f^
					25 µg L^−1^		100 µg L^−1^		
					Intra-day	Inter-day	Intra-day	Inter-day	
Acetochlor	0.55	1.83	2–1000	0.999	8.1	9.8	3.9	6.7	433 ± 7
Metalaxyl	0.23	0.76	2–1000	0.999	13.2	13.3	7.5	8.8	160 ± 1
Ametryn	0.11	0.35	2–1000	0.986	5.7	9.2	4.7	7.7	441 ± 6
Haloxyfop-*R*-methyl	0.12	0.41	2–1000	0.988	3.6	5.8	7.7	9.9	475 ± 8
Hexaconazole	0.23	0.78	2–1000	0.998	6.1	8.3	5.9	6.8	492 ± 10
Oxadiazon	0.22	0.74	2–1000	0.991	7.3	7.6	4.5	5.1	576 ± 10
Difenoconazole	0.29	0.95	2–1000	0.999	4.7	11.1	4.6	6.3	662 ± 11

a Limit of detection (S/N = 3) (µg L^−1^).

b Limit of quantification (S/N = 10) (µg L^−1^).

c Linear range (µg L^−1^).

d Coefficient of determination.

e Relative standard deviation for intra- (*n* = 7) and inter-day (*n* = 3) precisions.

f Enrichment factor ± standard deviation (*n* = 3).

### 3.8. Analysis of real samples

**Fig. 3:**
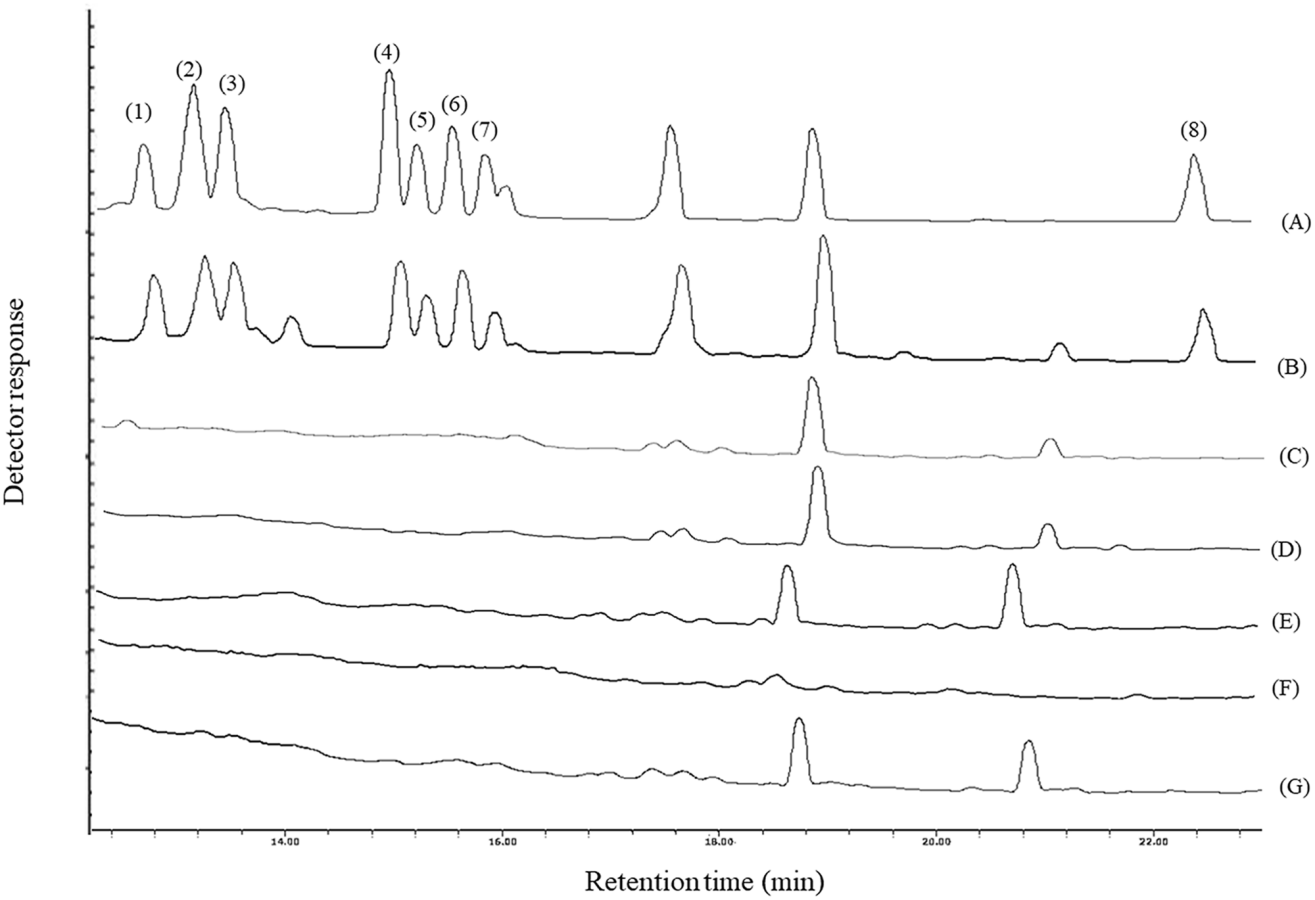
GC-FID chromatograms of standard solutions of the analytes (200 mg L^−1^ of each) in methanol (A), an aqueous solution (200 µg L^−1^ of each analyte) (B), Moldavian dragonhead (C), mint (D), pussy willow (E), cardamom (F), and cinnamon (G). The proposed method was applied on them, and 1 µL of the final organic phase was injected into GC-FID, except for chromatogram (A), in which it was injected directly. Peak identification: (1) acetochlor, (2) metalaxyl, (3) ametryn, (4) cetyl alcohol, (5) haloxyfop-*R*-methyl, (6) hexaconazole, (7) oxadiazon, and (8) difenoconazole.

To investigate the efficiency of this work, the pesticide contents of five distillates, including mint, Moldavian dragonhead, cardamom, pussy willow, and cinnamon, were analysed. None of the analytes was indicated in the samples. The related chromatograms are shown in Fig. 3. The subsequent matrix effect was studied with the added-found method. In Table 2, relative recovery data of the real samples spiked at three concentrations of 50, 100, and 250 µg L^−1^ (each pesticide) are reported with respect to deionized water spiked at the relevant concentration of the pesticide. The obtained data are in the range of 80–119%. As a result, the developed approach can be used for the detection of pesticides in these samples.

**Table 2:** Study of the matrix effect in distillate samples spiked at different concentrations

Analyte	Mean relative recovery				
	Mint	Moldavian dragonhead	Cardamom	Pussy willow	Cinnamon
**All samples were spiked with each analyte at a concentration of 50 µg L^−^** ^**1**^					
Acetochlor	95 ± 7	119 ± 9	117 ± 9	101 ± 8	82 ± 6
Metalaxyl	80 ± 10	80 ± 10	115 ± 15	80 ± 10	104 ± 13
Ametryn	116 ± 6	89 ± 5	118 ± 6	95 ± 5	113 ± 6
Haloxyfop-*R*-methyl	80 ± 4	90 ± 3	80 ± 2	86 ± 3	98 ± 3
Hexaconazole	86 ± 3	89 ± 5	96 ± 3	84 ± 5	116 ± 7
Oxadiazon	107 ± 7	92 ± 6	95 ± 5	106 ± 7	113 ± 8
Difenoconazole	89 ± 4	106 ± 4	80 ± 3	115 ± 5	83 ± 3

**All samples were spiked with each analyte at a concentration of 100 µg L^−^** ^**1**^					
Acetochlor	91 ± 7	80 ± 6	83 ± 6	109 ± 8	115 ± 9
Metalaxyl	104 ± 13	113 ± 14	113 ± 14	100 ± 13	112 ± 14
Ametryn	88 ± 5	103 ± 5	106 ± 6	83 ± 4	81 ± 4
Haloxyfop-*R*-methyl	80 ± 2	80 ± 2	89 ± 3	102 ± 3	93 ± 3
Hexaconazole	91 ± 5	97 ± 5	92 ± 5	88 ± 5	85 ± 3
Oxadiazon	103 ± 7	81 ± 5	113 ± 8	99 ± 7	87 ± 6
Difenoconazole	91 ± 4	89 ± 4	97 ± 4	104 ± 4	96 ± 4

**All samples were spiked with each analyte at a concentration of 250 µg L^−^** ^**1**^					
Acetochlor	114 ± 9	112 ± 14	118 ± 9	115 ± 9	83 ± 6
Metalaxyl	100 ± 13	97 ± 12	81 ± 10	96 ± 12	116 ± 15
Ametryn	109 ± 6	108 ± 6	84 ± 4	96 ± 5	91 ± 5
Haloxyfop-*R*-methyl	92 ± 3	80 ± 2	86 ± 3	100 ± 3	91 ± 3
Hexaconazole	98 ± 5	85 ± 5	108 ± 6	93 ± 5	86 ± 5
Oxadiazon	91 ± 6	80 ± 5	104 ± 7	91 ± 6	81 ± 5
Difenoconazole	80 ± 3	87 ± 4	117 ± 5	105 ± 4	92 ± 4

### 3.9. Comparison of the method with other approaches

Table 3 compares the method reported herein with some recently introduced methods for the analysis and preconcentration of pesticides. The LOD, LOQ, extraction time, RSD, *r*^2^, and EF values were compared with those from other reports. The LODs and LOQs in the method under study are less than those of most mentioned studies. The repeatability of the method is acceptable. This method has comparable LRs to the other methods. The EFs of this study are higher than or comparable to other approaches. The advantages of the expanded analytical method include high EFs, short extraction time, extensive LRs, low LODs and LOQs, and acceptable RSDs. To accurately determine the “micro-solvent” advantage, the total organic solvent consumption per extraction was calculated: 30 µL *n*-hexanol (homogenizing solvent) + 8 µL di-*n*-butyl ether (homogenizing solvent) = 38 µL per 10 mL sample. This is an order of magnitude lower than conventional DLLME methods, which typically use disperser and extraction solvents in milliliter and microliter scales, respectively, thereby significantly reducing organic waste and improving the environmental profile of the method. A comprehensive comparison (Table 3) demonstrated that the HLLME-GC-FID method offers several notable advantages over conventional techniques. First, it features very low organic solvent consumption, using only 38 µL of solvent per 10 mL sample, whereas DLLME or DSPE methods typically require milliliter-scale quantities of organic solvents. Second, this method provides high enrichment factors (160–662), which are comparable or even superior to many recent microextraction techniques for pesticides. Third, the extraction time is short (approximately 12 min), improving the speed of the analytical process. Fourth, the method requires only simple hardware, such as a glass test tube with a capillary tip, without the need for sorbents or specialized tools. Finally, it achieves lower limits of detection (LOD) in the range of 0.11–0.55 µg L^−1^, indicating high analytical sensitivity.

**Table 3:** Comparison of the proposed method with the other methods used for the preconcentration and determination of the target compounds

Method	Sample	LOD^a^	LOQ^b^	LR^c^	*r* ^2^ d	RSD^e^	EF^f^	Extraction time (min)	Ref.
PT-SPE-GC-FID^g^	Fruits	4–16	—	12–10 000	0.994–0.997	7.4–8.5	—	>11	36
HS-SDME-GC-FID^h^	Orange juice	0.97–0.98	—	10–100 000	0.991–0.995	4.2–4.8	280–315	>20	37
DSPE-DES-DLLME-GC-FID^i^	Fruit juices	0.30–0.70	—	0.70–4000	0.995≥	≤6.3	—	>13	38
CFPSE-DLLME-GC-FID^j^	Fruit juices	1.2–3.3	4.3–11.2	4.3–2000	—	<4.9	540–720	7	39
DSPE-DLLME-GC-FID^k^	Vegetable and fruit juices	0.31	—	1–500	—	4	309	14	40
HLLME-GC-FID^l^	Distillates	0.11–0.55	0.35–1.83	2–1000	0.986–0.999	3.6–13.2	160–662	12	Present work

a Limit of detection (µg L^−1^).

b Limit of quantification (µg L^−1^).

c Linear range (µg L^−1^).

d Coefficient of determination.

e Relative standard deviation (%).

f Enrichment factor.

g Pipette-tip-solid phase extraction-gas chromatography-flame ionization detection.

h Headspace single drop microextraction-gas chromatography-flame ionization detector.

i Dispersive solid phase extraction-deep eutectic solvent-dispersive liquid–liquid microextraction-gas chromatography-flame ionization detector.

j Continuous fabric phase sorptive extraction-dispersive liquid–liquid microextraction-gas chromatography-flame ionization detection.

k Dispersive solid phase extraction-dispersive liquid–liquid microextraction-gas chromatography-flame ionization detection.

l Homogeneous liquid–liquid microextraction-gas chromatography-flame ionization detection.

## 4 Conclusions

In this study, an HLLME method was investigated for the extraction and preconcentration of some pesticides from various distillate samples. In the proposed method, *n*-hexanol was used to prepare a homogeneous phase, and di-*n*-butyl ether was used as the homogeneity-breaking agent. The EF values obtained were in the range of 160–662. Also, the LOD and LOQ values were in the ranges of 0.11–0.55 and 0.35–1.83 µg L^−1^, respectively. The RSDs of the procedure for examining the precision of the method were acceptable. The advantages of this study include simplicity, high speed, cost-effectiveness, reliability, and high EFs. Also, because of the use of small volumes of organic solvents, this method can be environmentally friendly.

## Abbreviations

EF: Enrichment factor
GC-FID: Gas chromatography-flame ionization detector
HLLME: Homogeneous liquid–liquid microextraction
LOD: Limit of detection
LOQ: Limit of quantification
LR: Linear range
RSD: Relative standard deviation

## Author contributions

Mir Ali Farajzadeh (analytical methodology and editing of the manuscript). Mahsa Goreishizadeh (analytical analysis and writing the manuscript). Sanaz Barazandeh (analytical methodology and editing the manuscript). Mohammad Reza Afshar Mogaddam (analytical methodology).

## Conflicts of interest

The authors declare that they have no competing interest.

## Supplementary Material

RA-015-D5RA07135F-s001

## Data Availability

All data generated or analysed during this study are included in this published article. Supplementary information is available and the data were presented as figure. See DOI: https://doi.org/10.1039/d5ra07135f.
