# Supplementary material for: Development of a homogeneous liquid–liquid microextraction method using lighter than water solvents for the extraction of some pesticides from distillate samples followed by GC-FID determination
Source: RSC Adv. 2025 Nov 18;15(52):44083–92. doi: 10.1039/d5ra07135f (PMC12624386; doi:10.1039/d5ra07135f)
Supplement: RA-015-D5RA07135F-s001 [file RA-015-D5RA07135F-s001.pdf]

Supplementary Information (SI) for RSC Advances.
This journal is © The Royal Society of Chemistry 2025

Supplementary materials

Development of a homogenous liquid-liquid microextraction method using lighter than water solvents for the extraction of some pesticides from distillate samples followed by GC-FID determination

Mahsa Ghoreishizadeh [a], Mir Ali Farajzadeh [a,b*], Sanaz Barazandeh [a], Mohammad Reza Afshar Mogaddam [c,d,e]

[a] Department of Analytical Chemistry, Faculty of Chemistry, University of Tabriz, Tabriz, Iran

[b] Engineering Faculty, Near East University, 99138 Nicosia, North Cyprus, Mersin 10, Turkey

[c] Food and Drug Safety Research Center, Pharmaceutical Sciences Institute, Tabriz, University of Medical Sciences, Tabriz, Iran

[d] Research Center of New Material and Green Chemistry, Khazar University, 41 Mehseti Street, Baku AZ1096, Azerbaijan

[e] Pharmaceutical Sciences Institute, Tabriz, University of Medical Sciences, Tabriz, Iran

*Corresponding author: M.A. Farajzadeh

Tel.: +98 41 33393084

Fax: +98 41 33340191

E–mail address: mafarajzadeh@yahoo.com; mafarajzadeh@tabrizu.ac.ir

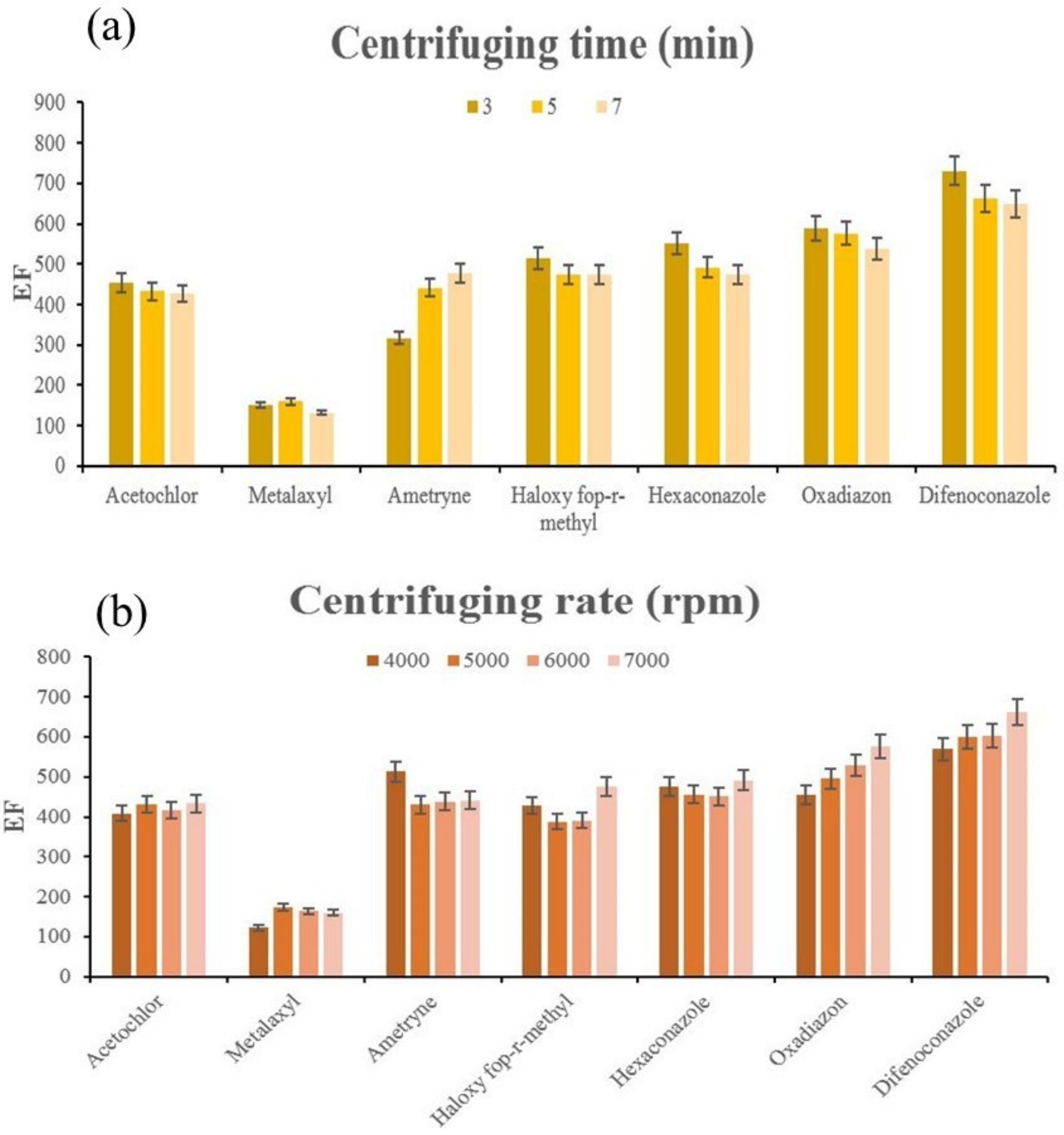

Fig. S1. Optimization of experimental parameters affecting the extraction efficiency of the proposed HLLME method:

(a) Centrifugation time.

Extraction conditions: are the same as those used in Figure 2e, except that pH 7.0 was used.

(b) Centrifugation rate.

Extraction conditions: are the same as those used in Figure S1a, except that centrifuging time was performed for 5 min.
